# Comprehensive characterization of ecological features and spatiotemporal distribution patterns of ticks in Shandong Province, China (2021–2023)

**DOI:** 10.1186/s13071-025-07055-9

**Published:** 2025-11-12

**Authors:** Yingnan Han, Hao Yin, Yan Liu, Xinyue Cao, Qintong Sun, Tong Cai, Yingchun Yang, Chenxin Han, Wenjie Liu, Hongmei Liu, Xuejun Wang

**Affiliations:** 1 Shandong Center for Disease Control and Prevention, Jinan, China; 2 Shandong Institute of Parasitic Diseases, Shandong First Medical University and Shandong Academy of Medical Sciences, Jining, 272033 Shandong People’s Republic of China; 3 School of Public Health, Shandong First Medical University and Shandong Academy of Medical Sciences, Jinan, China

**Keywords:** Tick fauna, Shandong Province, Spatiotemporal aggregation, Host association, Meteorological drivers

## Abstract

**Background:**

As a globally significant disease vector, ticks harbor diverse pathogens, occupy various ecological niches, and attach to a wide range of animal hosts. However, their distribution patterns in Shandong province remain poorly characterized.

**Methods:**

This study systematically investigated tick species composition, spatial–temporal distribution patterns, and environmental influencing factors, such as temperature, dewpoint temperature and precipitation, across 13 prefecture-level cities in Shandong province from 2021 to 2023.

**Results:**

*Haemaphysalis longicornis* was identified as the dominant species, accounting for over 90% of all collected ticks with widespread geographic distributions. Significant interannual variation in host-seeking tick densities was particularly, with 2021 recoding significantly higher values than 2022 and 2023. Densities were particularly elevated in suburban parks and unused grasslands compared with other habitat types. Host-specific analysis revealed that sheep exhibited significantly higher infestation rates and tick indices than dogs, cats, or cattle. Strong temporal aggregation patterns were observed in host-seeking and host collected tick counts from 2021 to 2023, with seasonal fluctuations peaking between March and June. While the host collected tick index remained stable across years, an unusual high tick density peak was recorded in Jinan in March 2021. Generalized Linear Mixed Model analysis indicated that mean air temperature was positively correlated with tick density, whereas mean dew point temperature showed a negative correlation.

**Conclusions:**

This study represents the first comprehensive ecological assessment of ticks in Shandong province and provides the first dataset for risk stratification and targeted prevention strategies for tick-borne diseases. These findings highlight the need to prioritize surveillance in rural habitats, focus on sheep as sentinel hosts, target spring as a high-risk period, and incorporate meteorological factors into early warning and prevention strategies.

**Graphical Abstract:**

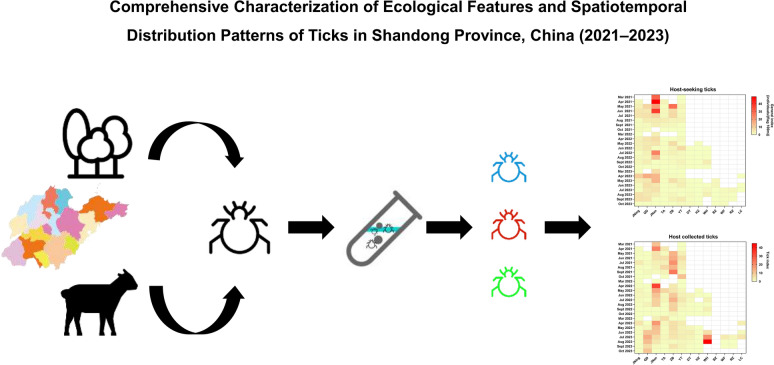

## Background

Ticks are significant vectors of zoonotic diseases, harboring a variety of pathogens that cause *Anaplasmosis*, *Babesiosis*, *Ehrlichiosis*, *Lyme borreliosis* and Severe fever with thrombocytopenia syndrome (SFTS) virus [1–5]. Ranked second only to mosquitoes among arthropod vectors in terms of public health impact, ticks pose a significant threat to human and animal health [6]. They inhabit a wide range of ecosystems, including forests, shrublands, grasslands, uncultivated pastures, and wastelands [7–10]. In terms of host selection, ticks parasitize the body surfaces of various vertebrates, including mammals, which serve as their primary hosts [11]. Similar to most arthropods, tick population dynamics and spatial distribution are influenced by complex interactions among microenvironmental factors (e.g., temperature and humidity) and regional meteorological variables (e.g., precipitation patterns) [7, 12]

Shandong Province, located in the lower Yellow River basin and bordered by the Bohai Sea and Yellow Sea to the east, shares administrative boundaries with Hebei, Henan, Anhui, and Jiangsu provinces. The region features a diverse geomorphology comprising central mountainous uplifts, northwestern and southwestern alluvial plains, and eastern hilly terrains that form a terrace-like configuration. Shandong’s warm-temperate monsoonal climate and heterogeneous landscape provide favorable ecological conditions for survivability and establishment of tick populations [13].

Tick-borne diseases pose significant public health concerns due to rising zoonotic incidence and impose substantial economic burdens on the livestock industry, resulting in a dual threat to health and agriculture [14, 15]. These concerns underscore the scientific need for vector surveillance to formulate regional control strategies. However, systematic research on tick ecology in Shandong province has not been carried out. To address this gap, a comprehensive field investigation was studied from 2021 to 2023 to characterize tick species composition, biogeographic distribution, and environmental factors (including meteorological and habitat variables) affecting their distributional dynamics across the province.

## Methods

### Study site and time

**Fig. 1:**
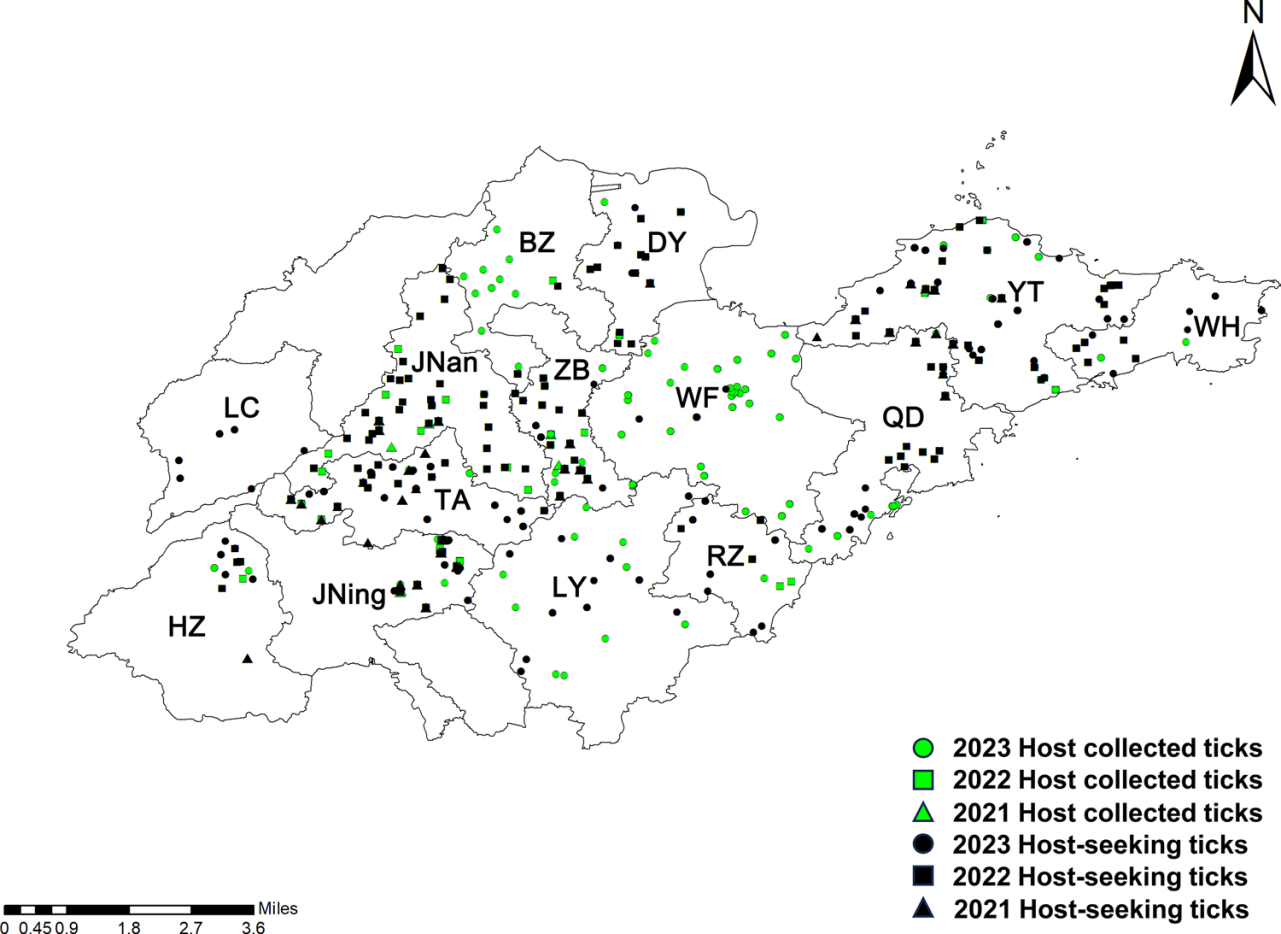
Location of tick sample sites in Shandong Province from 2021 to 2023. *JNan* Jinan, *TA* Taian, *JNing* Jining, *HZ* Heze, *LC* Liaocheng, *BZ* Binzhou, *DY* Dongying, *ZB* Zibo, *LY* Linyi, *QD* Qingdao, *YT* Yantai, *WH* Weihai, *RZ* Rizhao, *WF* Weifang

From 2021 to 2023, tick surveillance was conducted at multiple sites across Shandong province. In 2021, sampling points were established in Jinan, Qingdao, Jining, Tai’an, Zibo, and Yantai. In 2022, the sampling network was expanded to include Dongying, Heze, and Weihai. By 2023, additional sites in Liaocheng, Binzhou, Weifang, and Rizhao were incorporated, resulting in coverage of 13 administrative regions (Fig. 1, Table S1). If cities were designated as national-level monitoring sites, then at least two districts/counties were selected for tick surveys. Tick sampling was carried out between March and October, with peak activity observed from April to September. Monitoring extended into November in certain locations based on local climate conditions and operational requirements.

### Tick collection

Questing ticks were collected from vegetation using flags to sample host-seeking ticks from various habitats, including natural villages, farmlands, unused grasslands, woodlands, and public scenic areas such as urban parks, country parks, and forest parks. For the investigation of habitats, a stratified sampling method is generally adopted to select potential tick habitats conducive to tick collection as monitoring sites. These sites are placed across representative environments to ensure that the monitoring results accurately reflect the local distribution of ticks. At least one village was selected for each county defined as part of the tick surveys. Within the village periphery, randomly choose at least one habitat from farmland, unused grassland, or woodland. Additionally, included at least one location from public green spaces such as urban parks, country parks, and forest parks. Sampling conducted simultaneously once at each location. Light-colored flags measuring 90 × 65 cm were used to collect ticks from both ground-level and higher vegetation at selected locations. Tick density indices were calculated on the basis of the number of ticks captured at least per 500 m with a minimum time duration of 30 min. Preliminary tick identifications were recorded and number of ticks collected per time and/or distance [16].

Domestic animals in towns and rural settlements were inspected for attached ticks with at least 10 animals inspected in each village. Most of the hosts examined were cattle, sheep, dogs, and cats. In urban residential areas, pet hospitals and residential areas were selected to inspect hosts for attached ticks with at least 20 pets examined within county/district with most hosts consisting of dogs. Sampling conducted simultaneously once at each location.

Ticks collected from hosts, flags, or collection devices were removed using sterilized tweezers, placed into labeled collection tubes, and immediately sealed. Specimens from each site were pooled in site-specific tubes or labeled accordingly and transported to the laboratory for further analysis.

### Tick identification

Ticks were examined under a microscope in the laboratory to determine species and developmental stage. Morphological identification was performed according to established taxonomic keys and identification guidelines [17, 18].

### Meteorological data collection

Meteorological variables, including average air temperature (°C), average dew-point temperature (°C), and precipitation (mm), were collected for each prefecture-level city in Shandong Province. These data were sourced from the internationally exchanged datasets maintained under the protocols of the World Meteorological Organization (WMO) and are publicly accessible through the National Centers for Environmental Information (NCEI), National Oceanic and Atmospheric Administration (NOAA) (https://www.ncei.noaa.gov/cdo-web/).

### Data analysis

Monthly host-seeking ticks index and host collected ticks index were calculated for each prefecture-level city from 2021 to 2023 using Microsoft Excel statistical software package. Inter annual differences in host-seeking and host collected tick counts were assessed using the Kruskal–Wallis *H* test, with Dunn’s multiple comparisons test applied to host seeking tick density across years. One-way analysis of variance (ANOVA) was used to compare the annual mean density index across different habitats and to evaluate differences in the three-year mean tick prevalence rate and tick index among host collected ticks. Differences in host collected tick prevalence rate among host animal species were analyzed using the chi-square (*χ*^2^) test. The concentration index (*M*) was calculated to quantify the intra-annual aggregation of infestation. Generalized linear mixed models (GLMMs) were applied to assess the effect of meteorological variables on regional variation in host collected tick abundance.

Calculation formulas for each indicator:

$$ {\text{Density index }}\left( {{\text{individuals }}/ \, \left( {{\text{flag}} 100 {\text{m}}} \right)} \right)\, = \frac{{\text{Number of collected ticks}}}{{\text{Dragging distance}}} \times 100. $$

$$ {\text{Prevalence }}\left( \% \right)\, = \,\frac{{\text{Number of animals infested with ticks}}}{{\text{Total number of collected animals}}} \times { 1}00. $$

$$ {\text{Tick index}}\, = \,\frac{{\text{Number of collected ticks}}}{{\text{Total number of collected animals}}} $$

Concentration index:

$$ R_{x} = \frac{{r^{2} + r^{6} + r^{8} - r^{12} }}{2} + 3^{{{\raise0.7ex\hbox{$1$} \!\mathord{\left/ {\vphantom {1 2}}\right.\kern-0pt} \!\lower0.7ex\hbox{$2$}}}} \,\, \times \,\frac{{{\text{r}}^{3} { } + {\text{ r}}^{5} { } - {\text{ r}}^{9} { } - {\text{ r}}^{11} }}{2}\,\, + \,({\text{r}}_{{4}} - {\text{r}}_{{{1}0}} ). $$

$$ {\text{R}}_{{\text{y}}} \, = \frac{{{\text{r}}^{3} { } - {\text{ r}}^{{5{ }}} - {\text{ r}}^{9} { } + {\text{ r}}^{11} }}{2}\, + \,3^{{{\raise0.7ex\hbox{$1$} \!\mathord{\left/ {\vphantom {1 2}}\right.\kern-0pt} \!\lower0.7ex\hbox{$2$}}}} \, \times \,\frac{{{\text{r}}^{2} { } - {\text{ r}}^{6} { } - {\text{ r}}^{8} { } + {\text{ r}}^{12} }}{2}\, + \,({\text{r}}_{{1}} - {\text{r}}_{{7}} ). $$

$$ {\text{M = }}\,\sqrt {{\text{Rx}}^{2} + {\text{Ry}}^{2} } $$

*M* denotes the concentration index, and *r* represents the ratio of the number of ticks collected in a specific month to the total annual collected ticks, where the subscript indicates the month.

## Results

### Tick species composition

**Fig. 2:**
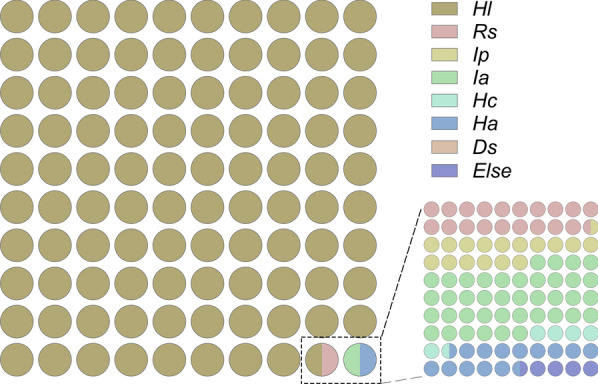
Composition and proportional distribution of tick species. *Hl Haemaphysalis longicornis, Rs Rhipicephalus sanguineus, Ip Ixodes persulcatus, Ds Dermacentor silvarum, Ha Haemaphysalis aponommoides, Hc Haemaphysalis companulata, Ia Ixodes acutitarsus*

From 2021 to 2023, a total of 33,141 ticks were collected from various habitats, with a mean density of 2.09 individuals per 100 m of drag-flag sampling. At the same time, 15,164 potential host animals were examined, among which 2164 animals were found to be infested, yielding a prevalence rate of 14.3% and a mean infestation intensity of 3.23 ticks per host (Table 1). Eight tick species were identified: *Haemaphysalis longicornis*, *Rhipicephalus sanguineus*, *Dermacentor silvarum*, *Ixodes acutitarsus*, *Haemaphysalis aponommoides*, *Ixodes persulcatus*, *Haemaphysalis companulata*, and several unidentified species. Among these, *H*. *longicornis* was dominant, comprising 98.5% of all collected specimens and exhibiting widespread distribution across all surveyed prefecture-level cities (Fig. 2, Table S2, Table 2).

**Table 1:** Distribution and proportional abundance of tick developmental stages across collection sites

	Vegetation			Domestic animals		
	2021	2022	2023	2021	2022	2023
Larvae	601 (8.6%)	1888 (18.5%)	2968 (18.6%)	90 (2.6%)	599 (7.5%)	824 (6.7%)
Nymphs	2166 (30.9%)	2994 (29.3%)	5060 (31.8%)	741 (21.5%)	2472 (30.8%)	2106 (17.1%)
Adult females	2436 (34.8%)	2840 (27.8%)	4736 (29.7%)	1625 (47.2%)	3571 (44.5%)	6389 (51.9%)
Adult males	1802 (25.7%)	2486 (24.4%)	3164 (19.9%)	984 (28.6%)	1378 (17.2%)	2993 (24.3%)
Total	7005	10,208	15,928	3440	8020	12,312

**Table 2:** Prefecture-level distribution of tick species in Shandong province

	Hl	Rs	Ip	Ds	Ha	Hc	Ia
JNan	+						
QD	+						
ZB	+	+			+		
YT	+						+
JNing	+	+					
TA	+	+		+	+		
HZ	+						
DY	+						
WH	+						
RZ	+						
BZ	+					+	
WF	+		+				
LY	+						
LC	+					+	

Presence ( +) denotes verified collection records

### Tick abundance and habitat-specific density

Host-seeking tick density indices displayed statistically significant interannual variation in Shandong Province. Mean density declined from 3.33 individuals / (flag 100 m) in 2021 to 2.19 in 2022 and 1.76 in 2023 (Kruskal–Wallis *H* test: *H* = 13.062, *df* = 2, *P* = 0.002). Post hoc analysis using Dunn’s test indicated that the 2021 density index differed significantly from both 2022 (Dunn test: *Z* = 3.27, *P* = 0.003) and 2023 (Dunn test: *Z* = 3.25, *P* = 0.004) (Table 3).

**Table 3:** Annual survey effort and tick collection efficiency by dragging method (2021–2023)

Year	Dragging time (min)	Dragging distance (m)	Number	Density index (individuals/ (flag 100 m)) ^a^	Kruskal–Wallis *H* test	Dunn’s test
2021	6944	105,226	3506	3.33	*H* = 13.062 *df* = 2 *P* = 0.002^*^	
2022	14,343	218,456.2	4783	2.19		*Z* = 34.32 *P* = 0.003^*^
2023	27,819	452,225	7942	1.76		*Z* = 32.54 *P* = 0.004^*^
Total	49,106	775,907.2	16,231	2.09		

^*^Statistically significant

^a^Density index = (number of collected ticks / dragging distance) × 100

**Fig. 3:**
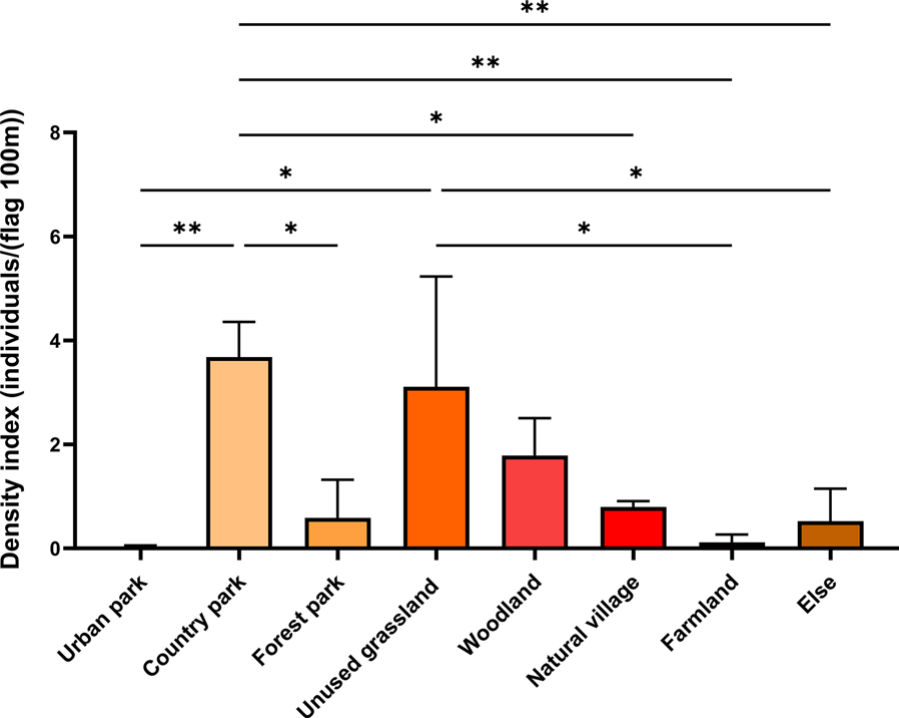
Differential tick density across habitat types. Error bars represent Mean ± SD; *P < 0.05, **P < 0.01

Tick surveillance encompassed seven habitat types: urban parks, country parks, forest parks, unused grasslands, woodlands, natural village, and farmlands. Across the three years, country parks and unused grasslands had significantly higher mean tick densities than other habitat types (Fig. 3, Table S3).

### Species, abundance, and density of host collected ticks

From 2021 to 2023, the mean density of host collected ticks in Shandong province remained relatively stable, with no statistically significant inter annual variation (Kruskal–Wallis H-test: *H* = 3.98, *df* = 2, *P* = 0.137). The mean number of ticks per host was 4.47 in 2021, 2.93 in 2022, and 3.08 in 2023 (Table 4).

**Table 4:** Surveillance of host collected ticks in Shandong province from 2021 to 2023

Year	Animals	Ticks	Infested animals	Prevalence^a^ (%)	Tick index^b^	Kruskal–Wallis test
2021	1059	4736	349	32.96	4.47	*H* = 3.98 *df* = 2 *P* = 0.137
2022	2503	7345	668	26.69	2.93	
2023	4020	12,399	1147	28.53	3.08	
Total	7579	24,480	2164	28.55	3.23	

^a^Prevalence (%) = (number of animals infested with ticks/total number of collected animals) × 100

^b^Tick index = number of collected ticks/total number of collected animals

**Fig. 4:**
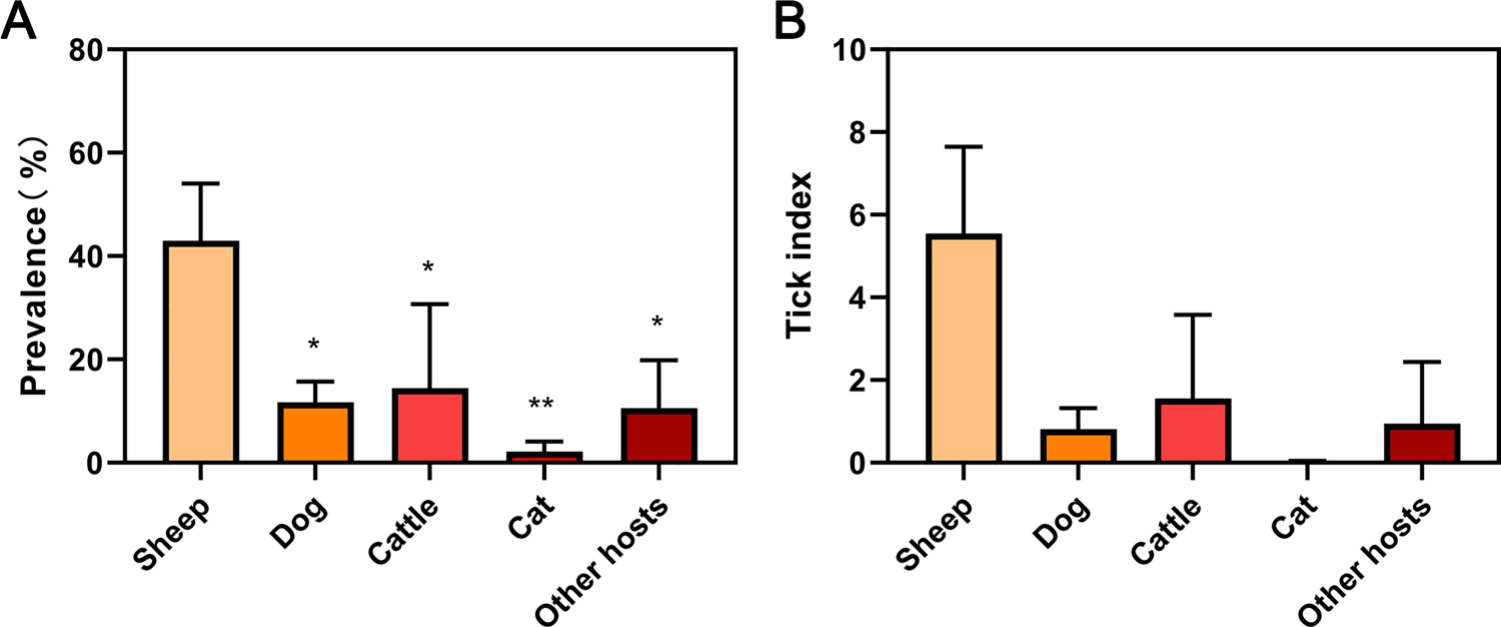
Host-specific variation in tick parasitism metrics. **A** Infestation prevalence (%, *P < 0.05, **P < 0.01). **B** Tick index (ticks/host). Mean ± SD shown

Host species investigated included sheep, cattle, dog, and cat. Including 4527 sheep, 2588 dogs, 287 cows, 101 cats, and 79 other hosts (ANOVA, *F*_(4, 10)_ = 7.38, *P* = 0.005). Among these, sheep showed the highest mean tick index over the three years (5.55), significantly exceeding those observed in dogs (*P* = 0.021), cats (*P* = 0.004), and cattle (*P* = 0.035) (Fig. 4, Table S4).

Across all tick specimens collected (*n* = 23,600), *Haemaphysalis longicornis* was the predominant species, accounting for 91.7%. Host-specific infestation analysis revealed sheep harbored the highest infestation intensity (mean = 3.94 ticks per host). Tick prevalence in sheep (32.4%) was significantly higher than in dogs (9.4%; Chi-square test, *χ2* = 510.25, *df* = 1, *P* < 0.001), cats (8.9%; Chi-square test, *χ2* = 25.16, *df* = 1, *P* < 0.001), and cattle (20.1%; Chi-square test, *χ2* = 18.57, *df* = 1, *P* < 0.001) (Table 5).

**Table 5:** Differential tick parasitism across host taxa

Host	Animals	Infested animals	Prevalence (%)	Ticks	Tick index	
Sheep	4830	1566	32.42	19,034	3.94	
Dog	2748	257	9.35	1918	0.7	*χ*^*2*^ = 510.25 *df* = 1 *P* < 0.001^*^
Cat	101	9	8.91	40	0.4	*χ*^*2*^ = 62.87 *df* = 1 *P* < 0.001^*^
Cattle	279	56	20.07	645	2.31	*χ*^*2*^ = 18.57 *df* = 1 *P* < 0.001^*^
Other hosts ^a^	23	4	17.39	4	0.17	/

^*^Statistically significant

^a^Other hosts: chickens, geese, pigs, hedgehogs, foxes, and other unrecorded hosts

### Seasonal fluctuations of ticks (2021–2023)

**Fig. 5:**
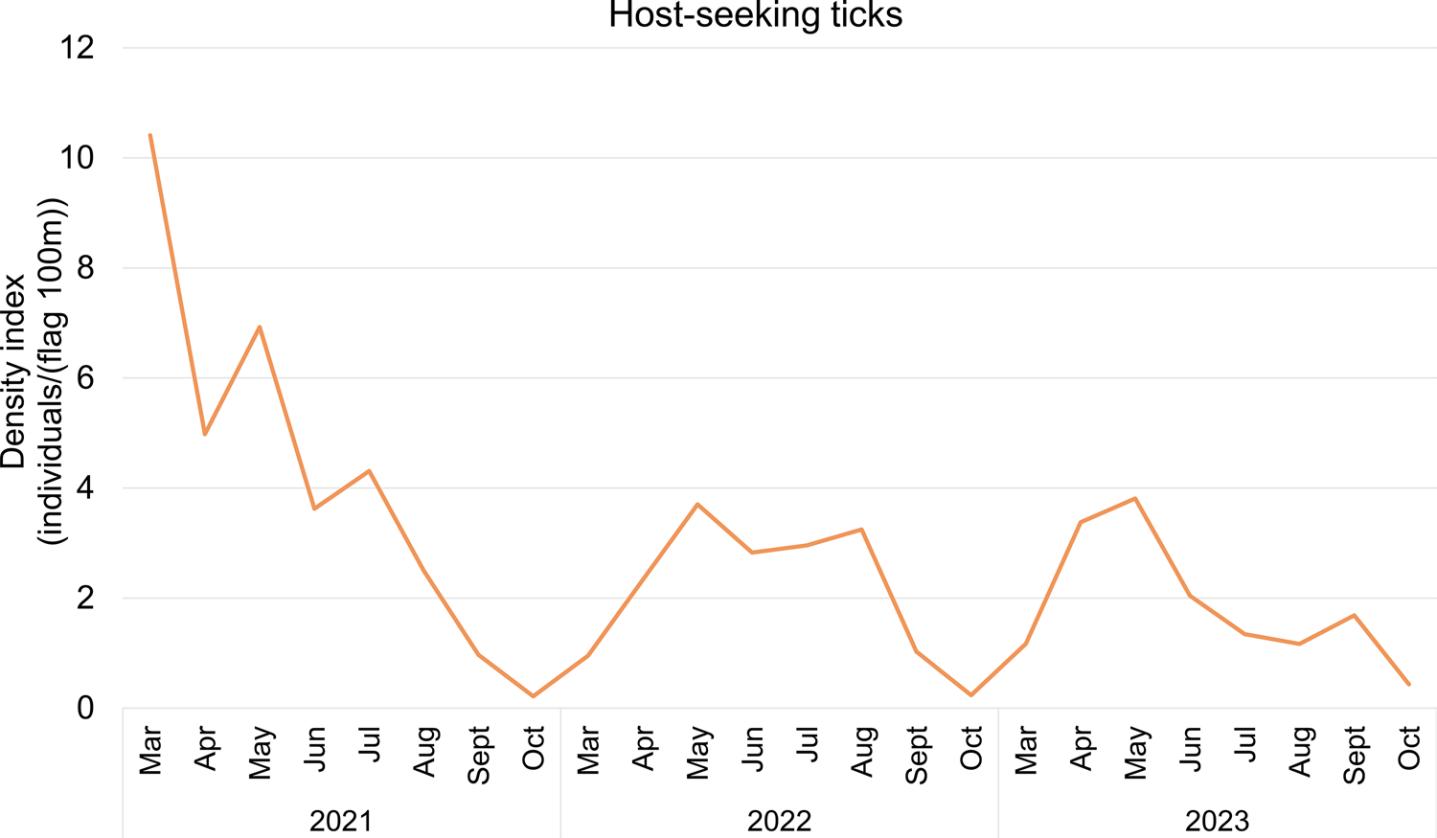
Time course of host-seeking tick density in Shandong Province, 2021–2023

**Fig. 6:**
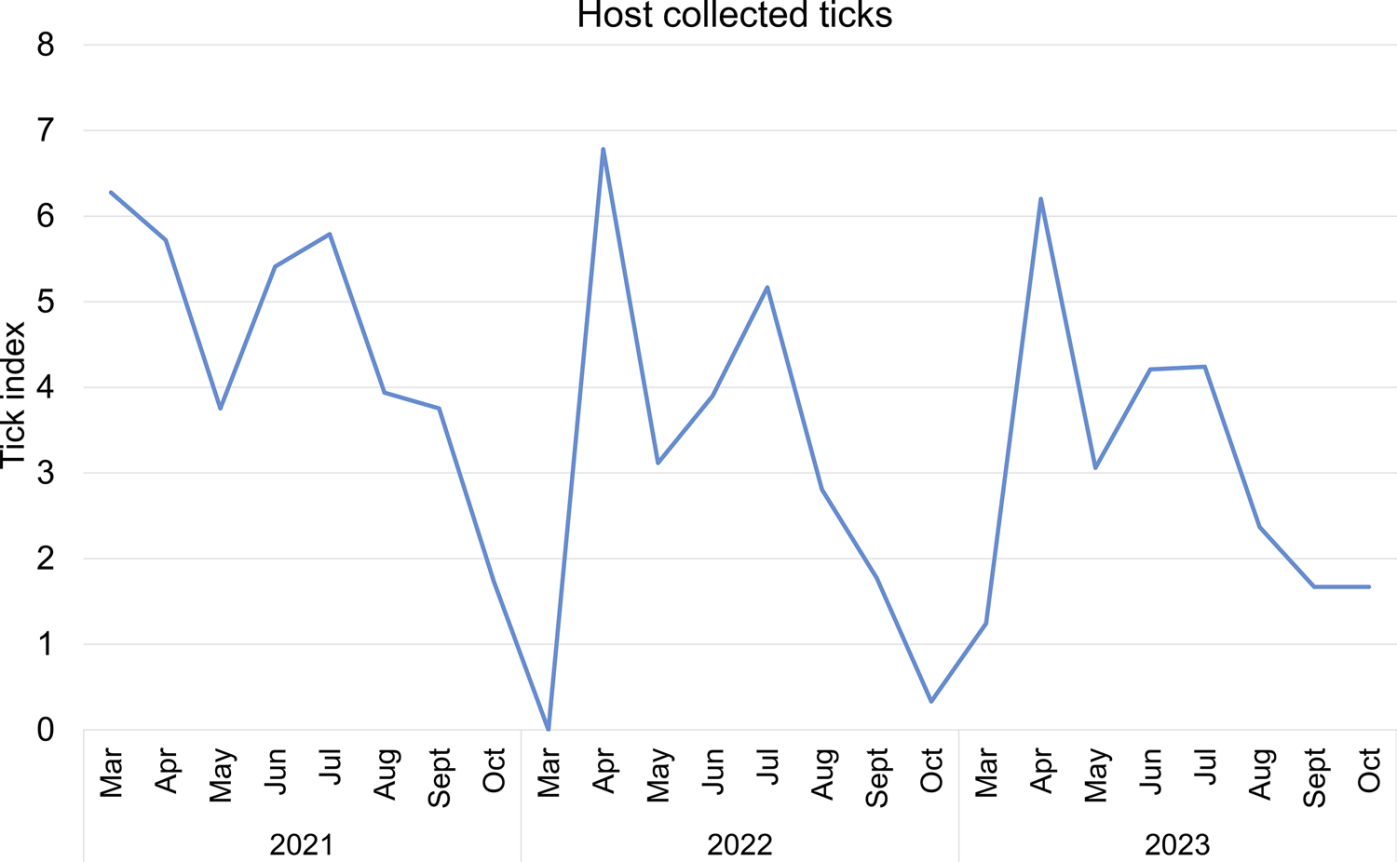
Time course of host collected tick index in Shandong Province, 2021–2023

Seasonal patterns indicated significant temporal aggregation of both host-seeking and host collected ticks, as shown by three-year average concentration indices of 0.62 and 0.68, respectively (Table 6). These values suggest consistent seasonal fluctuations in tick abundance. In 2021, an anomalous early-season surge in host-seeking tick density was observed, particularly from March to June, with the highest value recorded in March (10.41 individuals / (flag 100 m)) (Fig. 5, Table S5). Host collected tick activity also demonstrated seasonal dynamics. In 2022 and 2023, host collected tick numbers followed a bimodal distribution, with major peaks in April and July. However, in 2021, an atypical early peak occurred in March, similar to the pattern of host-seeking ticks (Fig. 6, Table S6).

**Table 6:** Concentration index of host collected ticks and host-seeking ticks in Shandong province (2021–2023)

	Year	*R*_*x*_	*R*_*y*_	*M*	*M* mean
Host collected ticks	2021	0.20	0.53	0.57	0.62
	2022	0.16	0.68	0.70	
	2023	0.22	0.56	0.60	
Host-seeking ticks	2021	0.37	0.62	0.73	0.68
	2022	0.08	0.72	0.73	
	2023	0.26	0.51	0.57

**Fig. 7:**
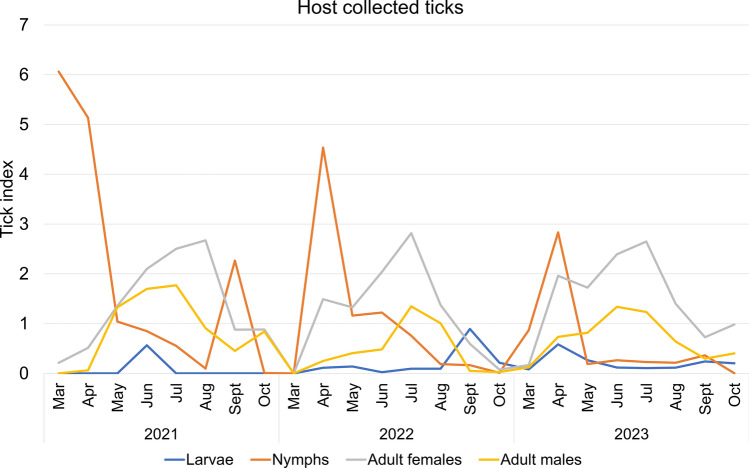
Stage-structured population dynamics of host collected ticks in Shandong (Mar 2021–Oct 2023)

**Fig. 8:**
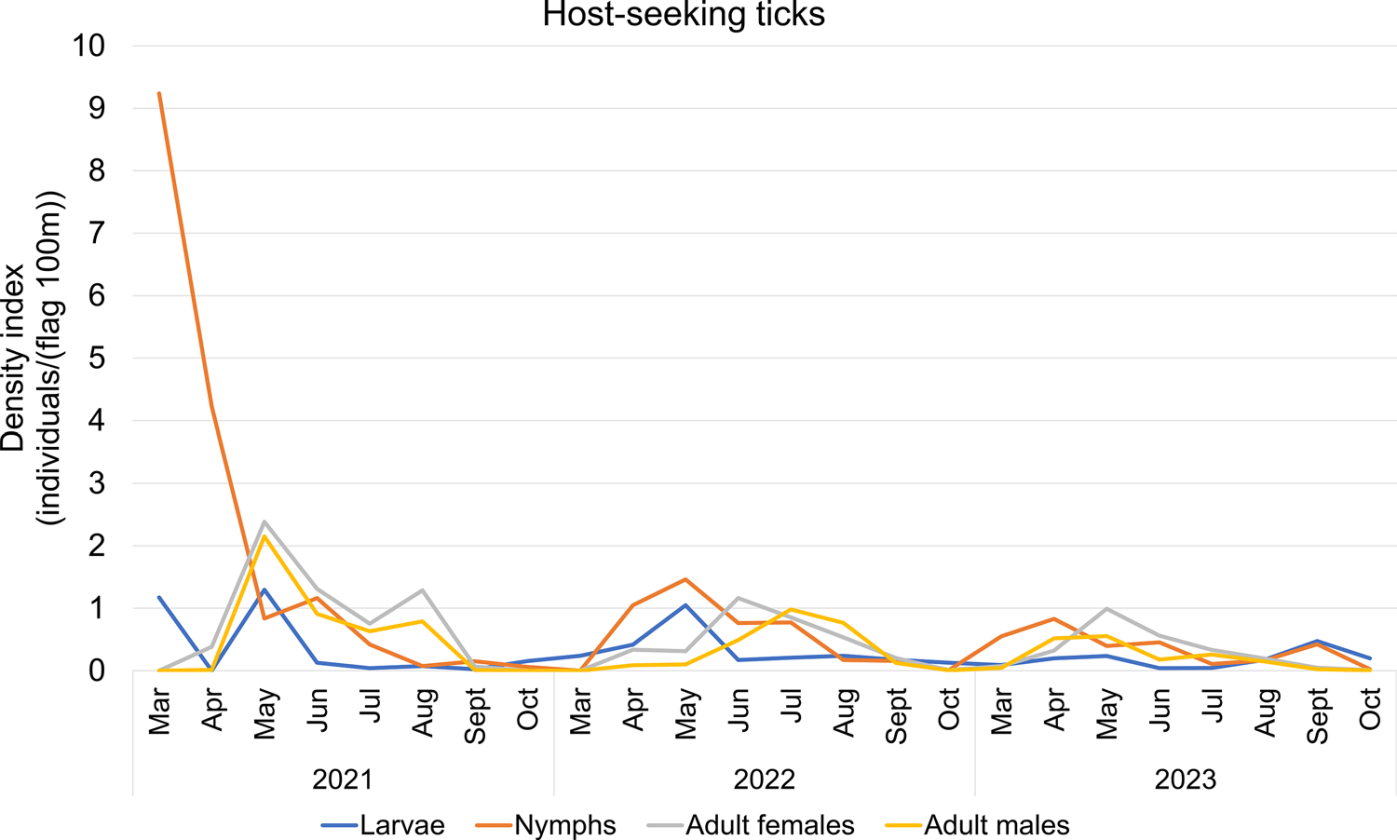
Stage-structured population dynamics of host-seeking ticks in Shandong (Mar 2021–Oct 2023)

Developmental stage-specific analysis revealed that variation in the number of host-seeking ticks at different stages of development also varied, with peaks in both larvae and nymph densities occurring earlier than adult female and adult male densities in 2022 and 2023. Nymphal densities peaked in April and declined thereafter, while adult female and male densities peaked in July and August. On the other hand, 2021 revealed unusually elevated nymph densities in March (9.24 individuals/(flag 100 m)), followed by April (4.22 individuals/(flag 100 m)), while adult female and male densities reached their peak in May (2.38 and 2.15 individuals/(flag 100 m), respectively) (Fig. 7, Table S7). While comparing host collected ticks at different developmental stages, the peak of nymphs in 2022 and 2023 appeared in April and gradually decreased. Nymphal also peaked in March 2021 (tick index = 6.06) and again showed an unexpected increase in September (2.27). In comparison, adult female and male host collected tick indices in all three years displayed a consistent pattern, with the peak in host collected tick density occurring in July and August, followed by a gradual decrease (Fig. 8, Table S8).

### Key factors driving abnormal distribution of host-seeking ticks in Jinan (2021)

**Fig. 9:**
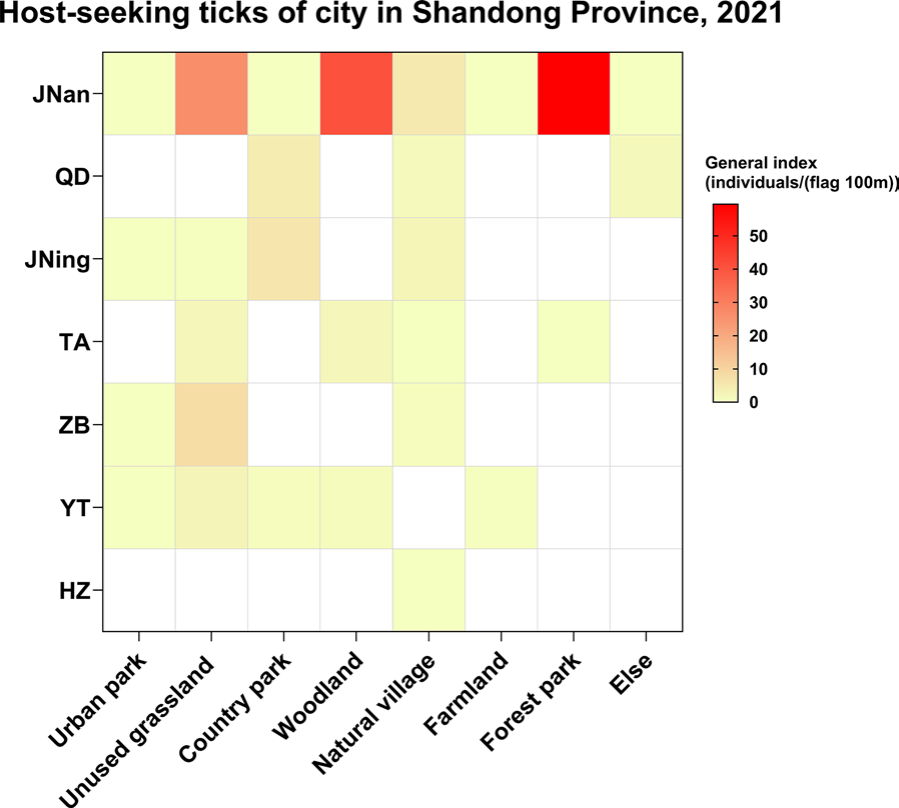
Spatial heterogeneity of host-seeking tick density across urban–rural gradients in Shandong, 2021

In 2021, tick density in Jinan was significantly elevated compared with other prefecture-level cities in Shandong province, particularly in unused grasslands (26.15 individuals/(flag 100 m)), woodlands (40.54 individuals / (flag 100 m)), and forest parks (59.46 individuals/(flag 100 m)). These values were significantly higher than those recorded in similar habitats elsewhere in Shandong province (Fig. 9, Table S9).

Analysis of annual average meteorological parameters across cities revealed that Jinan had the highest mean air temperature (15.92 ± 0.52 °C), along with significant variation in maximum temperature compared with other regions (Table 7). GLMM analysis identified mean air temperature (GLMM: *F* = 30.53*, df* = 14*, P* < 0.001, estimate = 0.48, SE = 0.09) and mean dew point temperature (GLMM: *F* = 24.99*, df* = 14*, P* < 0.001, estimate =  0.41, SE = 0.08) as significant predictors of tick density among the meteorological factors (Table 8).

**Table 7:** Climatic divergence across Shandong’s cities

	Temperature (℃)	Dewpoint temperature (℃)	Precipitation (mm)
BZ	14.61 ± 0.4	6.34 ± 0.21	67.18 ± 14.94
JNan	15.92 ± 0.52	5.22 ± 0.39	56.88 ± 22.02
JNing	15.71 ± 0.31	7.75 ± 0.36	68.04 ± 13.04
LC	15.27 ± 0.34	7.51 ± 0.43	62.58 ± 10.66
LY	15.46 ± 0.18	6.8 ± 0.71	78.49 ± 7.42
QD	14.1 ± 0.33	7.7 ± 0.21	73.45 ± 29.63
TA	7.19 ± 0.31	0.48 ± 0.28	113.72 ± 23.55
YT	13.63 ± 0.31	7.28 ± 0.16	57.16 ± 12.24
ZB	14.09 ± 0.4	5.19 ± 0.21	75.23 ± 14.94
WF	14.88 ± 0.44	5.93 ± 0.32	62.96 ± 22.74
WH	12.68 ± 0.3	7.99 ± 0.57	59.29 ± 14.22
RZ	14.55 ± 0.27	7.68 ± 0.55	67.16 ± 15.02
DZ	14.37 ± 0.28	6.49 ± 0.21	57.09 ± 11.25
HZ	15.77 ± 0.20	8.49 ± 0.29	70.42 ± 15.35
Kruskal–Wallis *H* test	36.48	35.97	
*df*	14	14	
*F*			1.45 (13, 28)
*P* value	0.002^*^	0.001^*^	0.20

^*^Statistically significant

**Table 8:** GLMM for temperature and dewpoint effects

	*F*	*df*	*P*	Estimate	SE	95% Confidence Interval	
						Lower bound	Upper bound
Temperature (℃)	30.53	14	0.00*	0.48	0.09	0.30	0.67
Dewpoint temperature (℃)	24.99	14	0.00*	0.41	0.08	0.58	0.23

^*^Statistically significant

## Discussion

Globally, ticks represent the second most significant arthropod vectors of human and animal pathogens, surpassed only by mosquitoes [2, 6]. China is home to 117 documented tick species, of which 60 (51%) are confirmed vectors of infectious agents and occupy distinct biogeographical niches [19]. This study conducted a systematic spatiotemporal investigation of both host-seeking and host-attached tick populations across 13 prefecture-level municipalities in Shandong Province between 2021 and 2023, employing standardized transect sampling and morphological identification protocols. A total of seven tick species were identified, with *H*. *longicornis* as the dominant species collected from all habitat types and hosts. These findings are consistent with epidemiological surveys conducted in adjacent provinces such as Henan [20, 21]. The greatest diversity of tick species was found in Tai’an. However, the sampling methodology may have influenced species representation. The reliance on drag-cloth sampling and livestock hosts could overestimate actively host-seeking ticks such as *H*. *longicornis* adults [16]. Furthermore, the host-seeking behaviors and microhabitat preferences of ticks exhibit significant diel and seasonal variations [22, 23]. In addition, the absence of wildlife host sampling (e.g., rodents, birds) may omit tick species with narrow host preferences. Future studies should integrate wildlife host sampling and microhabitat-specific collection methods to address these limitations. Such approaches would enhance the representativeness of tick community assessments and associated pathogen risks. The ecological dominance of *H*. *longicornis*, a primary vector of SFTSV and other zoonotic pathogens, underscores considerable tick-borne disease transmission risk in Shandong province, similar to its recognized epidemiological role in comparable temperate East Asian regions [24–27]. Additional tick species also warrant further attention regarding their disease risks. For example, *Ixodes acutitarsus* can transmit *B*. *burgdorferi* sensu lato; *Rhipicephalus sanguineus* serves as a vector for spotted fever group *rickettsiae*, *Anaplasma,* and the South African strain of *Ehrlichia canis* [28, 29].

Quantitative analysis revealed significant interannual variation in host-seeking tick populations, with 2021 exhibiting a mean density index of 3.33 individuals / (flag 100 m), 1.8 times higher than in 2022 (2.19) and 2023 (1.76) (Kruskal–Wallis *H* test, *P* < 0.01). Habitat-specific comparisons indicated that country parks and unused grasslands supported significantly higher tick densities than other land types. These differences may be due to microclimatic conditions such as soil moisture, temperature, vegetation cover, and host animal activity [7, 30–32]. In Europe, tick abundance is higher in forests than in open grasslands [33]. Similarly, in China’s grassland environments, grazing affects arthropod abundance, and changes in arthropod abundance are closely linked to grazing-induced vegetation height [34]. Compared with grazed areas (which are typically regularly grazed short meadows), unused grasslands with taller vegetation provide a more favorable habitat for tick survival and reproduction [7]. On the other hand, tick densities were significantly lower in urban parks and farmlands, likely owing to anthropogenic disturbances that degrade habitat quality and suppress tick survival [35].

No significant interannual differences regarding host association were observed in host collected tick indices. However, when comparing tick prevalence and tick indices between host animals, sheep consistently showed higher tick prevalence and infestation intensity than dogs, cats, or cattle, corroborating regional zoonotic surveillance data identifying sheep as keystone reservoir hosts in local tick-borne disease cycles [36, 37]. The differences in infestation rates may depend on the extent to which preventive measures are applied and the bioecological characteristics of tick species, which are closely related to climatic conditions and the specific animal rearing patterns in each region [38]. *H*. *longicornis* displays limited dispersal capacity, typically adopting a sessile lifestyle characterized by ambush predation tactics in microhabitats frequented by hosts [39]. In summer, flocks of sheep are mostly raised by grazing. The coverage rate of shrub grassland is the most important vegetation feature affecting the distribution of the long-horned tick [40]. Current research indicates that the head is the site with the highest tick count on sheep and goats [41]. This is likely because sheep foraging in the wild become infested with ticks while grazing. Free-ranging goats and sheep, unlike confined cattle, tend to graze in marginal habitats such as rural parks and heathlands, therefore increasing their exposure to tick-infested environments [42]. Moreover, intensive small ruminant farming systems create enclosed, humid microclimates conducive to tick breeding within human settlements [43]. These enclosures should be prioritized in tick control interventions, while protective measures are warranted for livestock handlers and agricultural workers to minimize occupational exposure to *H*. *longicornis*. During disease epidemic seasons, spraying insecticides can effectively reduce the density of host collected ticks, thereby lowering disease transmission risks. It is recommended to use specialized acaricides to enhance efficacy and delay resistance. Additionally, vegetation management should be strengthened to minimize tick breeding habitats [44].

From 2021 to 2023, both host-seeking and host-associated tick populations in Shandong province displayed noticeable seasonal trends, with an anomalous increase in tick activity observed in March 2021. Most of the specimens collected during this period were from Jinan City, the capital of Shandong province. Characterized by a temperate monsoon climate, Jinan experiences relatively high temperatures and humidity during spring and summer seasons, which align with ticks’ preference for damp, shaded vegetation environments. The city’s terrain displays a notable north–south elevation gradient influenced by the Taishan Mountain Range, with southern regions dominated by mountainous and hilly landscapes featuring comparatively high forest coverage rates. These mountainous areas create microclimatic conditions (including elevated humidity and moderated shade) that significantly enhance tick survival and reproductive success [45]. Additionally, during the winter of December 2020 to February 2021, a significant warming trend was observed in North China after 13 January, accompanied by a noticeable increase in precipitation [46]. *H*. *longicornis* primarily overwinters in the nymph and adult stages. Studies indicate that nymphs become active when temperatures fluctuate above 10 °C in March, while adults require more stable warm conditions, peaking in activity from April to June [47, 48]. Consequently, these conditions caused a surge, particularly in nymph populations, across Shandong Province during early 2021. Developmental stage-specific analyses indicated significantly elevated densities of host-seeking ticks during March, April, and May of 2021 compared with corresponding periods in subsequent years. Climatic variables such as ambient temperature, relative humidity, and precipitation are known to regulate tick development, survival, and spatial distribution, thus affecting the risk of tick-borne disease transmission [7, 49]. GLMM analysis of meteorological parameters affecting inter-regional variation in host-seeking tick densities across prefectures and months during 2021 revealed that mean atmospheric temperature and dew point were significantly associated with regional variations in host-seeking tick densities. Ticks exhibit a high dependence on ambient temperature for all life activities, a characteristic that results in their population dynamics being strongly correlated with atmospheric temperature fluctuations. Temperature critically regulates tick survival, development, and reproductive success throughout their life history, from egg development to adulthood [50, 51]. Dew point temperature, defined as the threshold at which air becomes saturated with water vapor, serves as an indicator of ambient moisture content. However, the effects of atmospheric humidity on tick behavior remain controversial. Recent research demonstrates divergent perspectives while some studies suggest that elevated humidity disrupts osmoregulatory homeostasis and reduces tick activity due to hydric stress [29], others report improved reproductive output in *H*. *longicornis* populations under high-humidity conditions [52]. Therefore, both excessively high and low humidity can have an impact on the growth and development of ticks.

## Conclusions

In summary, this study provides a comprehensive assessment of ixodid tick community structure, seasonal fluctuations, and habitat- and host-specific distribution across Shandong Province from 2021 to 2023. *H*. *longicornis* was the predominant species identified, with the highest densities recorded in country parks and grasslands and the highest host infestation intensity observed in sheep. The characteristic seasonal activity, particularly the anomalous peak of host-seeking tick densities observed in spring 2021, highlights the importance of intensified vector surveillance during high-risk months (March–June). Preliminary multivariate analyses identified mean temperature and dew point as key meteorological determinants of habitat-specific tick abundance. These findings contribute to the ecological framework for regional vector control strategies. Future research should incorporate molecular screening for pathogens and improve spatial modeling efforts by integrating data on host movement, anthropogenic land-use change, acaricide treatments, chemical and vegetation modifications, vaccines, a more integrated pest management approach, and microclimatic variation to improve zoonotic risk assessments in the region.

## Data Availability

All data generated or analyzed during this study are included in this published article and its additional files.

## Abbreviations

ANOVA: One-way analysis of variance
GLMMs: Generalized linear mixed models
JNan: Jinan
TA: Taian
JNing: Jining
HZ: Heze
LC: Liaocheng
BZ: Binzhou
DY: Dongying
ZB: Zibo
LY: Linyi
QD: Qingdao
YT: Yantai
WH: Weihai
RZ: Rizhao
WF: Weifang
Hl: *Haemaphysalis longicornis*
Rs: *Rhipicephalus sanguineus*
Ip: *Ixodes persulcatus*
Ds: *Dermacentor silvarum*
Ha: *Haemaphysalis aponommoides*
Hc: *Haemaphysalis companulata*
Ia: *Ixodes acutitarsus*
